# Financial Barriers to Expanded Birth Center Access in New Jersey: A Qualitative Thematic Analysis

**DOI:** 10.1111/jmwh.13732

**Published:** 2025-01-10

**Authors:** Rebecca H. Ofrane, Slawa Rokicki, Leslie Kantor, Julie Blumenfeld

**Affiliations:** ^1^ Department of Public Health Montclair State University, College for Community Health Montclair New Jersey; ^2^ Rutgers University School of Public Health Piscataway New Jersey; ^3^ Rutgers University School of Nursing New Brunswick New Jersey

**Keywords:** birth centers, birth equity, community birth, Medicaid reimbursement, midwifery workforce

## Abstract

**Introduction:**

Birth centers are an underused care setting with potential to improve birth experience and satisfaction. Both hospital‐based and freestanding birth centers operate with the midwifery model of care that focuses on safe, low‐intervention physiologic birth experiences for healthy, low‐risk pregnant people. However, financial barriers limit freestanding birth center sustainability and accessibility in New Jersey, especially for traditionally marginalized populations. This qualitative study explores the financial barriers faced by freestanding birth centers in order to expand access and choice for pregnant people in New Jersey.

**Methods:**

Semistructured interviews were conducted with participants from 4 sectors: (1) birth center or health system, (2) policy‐adjacent philanthropy or research, (3) state departments, and (4) health insurance. Coding and analysis followed a reflexive thematic analysis process, resulting in the identification of 4 financial barriers to birth center access.

**Results:**

Facility Medicaid reimbursement rates are a primary barrier for birth centers, along with startup and operating costs and, more indirectly, low supply of midwives and low patient demand for birth center care.

**Discussion:**

New Jersey is well‐positioned to enact critical policies and programs that can improve out‐of‐hospital birth center access, based on the findings and recommendations from this research. Other states can follow suit in pursuit of solutions to improve maternal health access and equitable birth center sustainability.

## INTRODUCTION

Positive birth experience and satisfaction with perinatal care are critical indicators of outcomes, and freestanding birth centers offer promising improvements for low‐risk pregnant people.^1^, ^2^ Among Black and Hispanic birthing people, roughly 30% report mistreatment during perinatal care broadly, and almost 40% report discrimination during maternity care, where it can be seen as disempowering and alienating.^3^, ^4^ Yet when looking specifically at birth center care, superior quality experience is evidenced; of those cared for in a freestanding birth center, 85% report being satisfied or extremely satisfied with their birth experience.^5^ The tenets of birth center care are centered around the experience of the birthing person and family, midwifery‐led, and available for healthy, low‐risk birthing people.^6^, ^7^, ^8^ Although nationally in the United States there were 415 freestanding birth centers operating in 2024, only 0.64% of births took place in these settings.^7^ In Declercq and colleagues’ survey of mothers who had given birth in hospitals, 64% expressed an interest in a birth center for a future birth, with one‐quarter saying they definitely wanted a birth center birth.^9^ Yet birth center access and use remain low. More than 90% of pregnancies are categorized as low risk, yet only 2% of pregnant people in the United States give birth outside of the hospital, suggesting that more low‐risk pregnant people could safely birth at birth centers, preserving hospital care for patients at higher risk.^10^, ^11^ Choice in birth setting can improve patients’ experience and autonomy. As described in the 2020 National Academies of Sciences, Engineering and Medicine Consensus Report titled *Birth Settings in America*, “women want safety, freedom of choice in birth setting and provider, choice among care practices, and respectful treatment.”^6(p 261)^ The authors acknowledge the different models of birth center care and community birth, and this study focuses on the American Association of Birth Centers definition of freestanding (ie, out of hospital) birth centers.^12^

**QUICK POINTS**

✦ Birth centers are a safe and effective birth setting for individuals with low‐risk pregnancies, but financial barriers prevent widespread accessibility in New Jersey and elsewhere.
✦ Medicaid reimbursement rates are a primary barrier for birth centers, along with startup and operating costs, limited midwifery workforce availability, patient awareness, and general knowledge of birth center safety.
✦ New Jersey is well positioned to enact critical legislative and practice changes that would serve as exemplary solutions for other states to follow suit, improving birth center sustainability and access, and therefore improved choice and birth experience nationwide.

### Background

Maternity care financing is influenced by Medicaid programming, representing the largest payer for births among women of color nationally and in New Jersey.^13^ The 2010 Affordable Care Act mandated birth center coverage in Medicaid, and although 41 states technically have Medicaid reimbursement for birth center providers and facility fees, actual payment implementation is inadequate and often covers only fee‐for‐service and not managed care organizations (MCOs).^14^ Medicaid programs pay approximately half the amount that private health plans pay for childbirth‐related expenses.^15^ Clinical facilities must then rely on a variety of payer mixes to stay solvent. Moreover, birth centers are often limited in their ability to provide enhanced or related services as compared with the diversified billable services provided by hospitals. Although corporate structures vary, most birth centers function as nonprofit organizations, with limited funding variability, which further hampers the financial sustainability of many centers.^16^ As of November 2023, there were 4 accredited freestanding birth centers in New Jersey, and of those, only 3 contracted with Medicaid fee‐for‐service or any of the 5 New Jersey MCOs and in limited contractual capacities.

The largest study to date relevant to birth center success and viability has been Strong Start and its accompanying evaluation.^8^, ^17^ Strong Start tested 3 outpatient models of *enhanced* prenatal care for Medicaid beneficiaries, including freestanding birth centers; the initiative served 45,999 pregnant people.^8^ The evaluation of Strong Start birth centers found many barriers to birth center access by Medicaid members, despite their strong interest and effective services.^8^ These barriers included (1) inadequate reimbursement for midwives and birth center services, (2) inability to contract directly with MCOs, (3) coverage limitations for services, (4) limited ability to participate in delivery system reforms, (5) state and local licensure laws, and (6) malpractice premiums and certification costs.^8^, ^15^, ^18^

Birth centers experience financial instability while providing high‐quality care for low‐risk pregnant people at lower costs. It has been estimated that if just 10% more US women give birth at home or in freestanding birth centers, health systems and insurers could save more than $10.8 billion per year.^19^ These cost analysis findings are consistent across studies and locations both in the United States and in other developed countries.^7^, ^20^, ^21^, ^22^, ^23^, ^24^

Despite being a promising model of care to improve outcomes, experience, and cost, access to birth centers in New Jersey remains limited. Other state‐specific analyses show similar issues of inadequate insurance reimbursement for birth centers and poor integration into the traditional health care system.^15^, ^25^ The financial barriers to birth center operations and integration into the birth ecosystem, and in turn patient access, point directly to a gap in research explored in this qualitative analysis. The purpose of this study was to identify the barriers to operating and sustaining freestanding birth centers in New Jersey, which in turn would expand access to more birthing people.

## METHODS

Exploratory qualitative methods with semistructured interviews were used for primary data collection. As the research question focused on barriers among a unique and small number of potential informants, this methodology was an appropriate choice. Coding and analysis followed a reflexive thematic analysis process,^26^, ^27^ informed by the research question and theoretical framework. More specifically, we followed Braun and Clark's nonlinear reflexive thematic analysis phases: familiarization, coding, initial theme generation, development, refinement, and writing.^26^ The Standards for Reporting Qualitative Research were followed in preparing this article (Supporting Information: Appendix S1).^28^

### Study Population and Recruitment

Because the study involved policy research and participant interviews about professional experience with systems and policies, the Rutgers University Institutional Review Board determined it to be nonhuman subject research (Pro2023000602). Interview participants were identified using stratified purposeful and snowball sampling, from 4 target sectors: birth center or health systems, policy‐adjacent sectors like philanthropy or research, state departments or offices responsible for policy or licensing, and health insurance‐payers. Invitations were extended to leadership of the operating birth centers in the state, and to experts in each sector based on the authors’ networks and experience, and continued through snowball sampling. Interviews were conducted with a range of professionals in roles that connect to the delivery of birth center care, birth center staff, midwives who practice at birth centers, collaborating physicians, hospital and health care administrators, Medicaid and MCO administrators, and other experts on relevant state policy, birth center care delivery models, and state licensure processes.

### Interviews and Coding

A total of 16 key informants representing the 4 sectors were interviewed (out of more than 20 invited participants) throughout May and June 2023. The primary investigator (PI) conducted the interviews using a semistructured interview format, leading to saturation of thematic content. Interviews focused on participants’ professional views on birth center licensing and operations, demographics of patients and staff, barriers to access, and types of insurance accepted. Interviews occurred virtually on Zoom, were recorded and transcribed using Zoom and Otter.ai, and were reviewed using an immersive process of simultaneously listening, reading, and correcting.

An integrated approach was used to develop code structure, both inductive development of codes as well as a deductive organization framework.^29^ All transcribed interviews were anonymously coded by the PI and a research assistant in Dedoose (version 9.0.107; intercoder reliability rate of 0.75). Conflicts were resolved using consensus coding and one‐to‐one review.^30^ Before resolution, there were 699 excerpts and 1419 code applications across both coders. After resolution and deduplication, the final data set consisted of 444 excerpts and 883 code applications.

### Thematic Analysis

After data familiarization and coding, analysis continued with reflexive thematic analysis phases: initial theme generation, development, refinement, and writing. The PI then reviewed coded interview excerpts reflexively from different perspectives, including key informant sector, years of experience, and observed or stated race and ethnicity, as well as the excerpts’ context related to pregnant individuals or to the regulatory or payer system. Code application patterns were analyzed and regrouped from the original codebook groupings into thematic groupings based on code frequency. Data analysis continued with identifying keywords in context that addressed the research question and reviewing excerpts related to financial drivers of birth center access.

### Reflexivity

The PI who designed the study and collected and analyzed the data has worked as a doula in both hospitals and birth centers; author J.B. is a certified nurse‐midwife and has practiced clinically for 2 decades. Authors L.K. and S.R. are established researchers with expertise in maternal and reproductive health policy. The process of reflexive thematic analysis includes considering your perspective to the data at each stage of the analysis, and the authors (who all identify as White women) acknowledge the limited involvement from participants of color in the interviews. Through this reflexive thematic analysis process, the PI identified the themes and barriers, and all other authors collectively reviewed and commented on the findings.

## RESULTS

Of the 16 interview participants, 8 represented the birth center or health system sector, 4 were in policy‐adjacent sectors like philanthropy or research, 2 were from state departments or offices, and 2 were from the health insurance‐payer sector. Of the 4 freestanding birth centers in New Jersey at the time of the study, 2 responded; 4 of the interviewees represented those centers. Throughout the results, interview excerpts are attributed to participants by their grouped sector. See Table 1 for participant demographic characteristics.

**Table 1:** Demographic Characteristics of Key Informants for Birth Benter Financial Barriers

Characteristics (N = 16)	Value
**Observed or stated race/ethnicity, n (%)**	
White, non‐Hispanic	12 (75)
Asian, non‐Hispanic	2 (13)
Hispanic	1 (6)
Black, non‐Hispanic	1 (6)
**Years of relevant experience in their field, n (%)**	
≤5	4 (25)
6‐15	4 (25)
>15	8 (50)
**Professional sector, n (%)**	
Birth center or health system	8 (50)
Policy‐adjacent philanthropy or research	4 (25)
State department or office	2 (12.5)
Health insurance or Medicaid payer	2 (12.5)

The financial barriers for birth centers to expand access to pregnant people in New Jersey were categorized as direct and indirect. Direct financial barriers included inadequate Medicaid facility reimbursement rates along with startup and ongoing operating costs. Indirect financial barriers included low supply of midwives and low but growing patient demand. These resulting barriers and interview excerpts are discussed below and summarized in Table 2.

**Table 2:** Summarized Financial Barriers to Birth Center Access, Exemplified by Interview Excerpts

Barriers and Interview Excerpts	Interview Participant's Sector
**Barrier 1: Inadequate facility reimbursement through Medicaid**	
The reimbursement rates aren't the best for Medicaid, it's sometimes more financially sound or more financially beneficial [for a facility] to just not accept Medicaid.	State official
We don't have contracts with all the Medicaid MCOs because some of them are offering us such low, low reimbursement that it wouldn't even pay for, you know, the sheets.	Birth center or hospital
I think if the regs would change… so that all birth centers could afford to accept Medicaid, I would like to see that so that everyone has access.	Birth center or hospital
**Barrier 2: Startup and operating costs**	
With the New Jersey regs, you can't like… like older birth centers in New Jersey were in an old Victorian home… that would never fly now with the ambulatory regulations. Like you have to have the hallway be a certain size, it costs so much money just to be able to meet the ambulatory requirements. We're not like other states where we could just have a birth center in a home anymore.	Birth center or hospital
This project was 5 million, who has that kind of money? Certainly not a midwifery practice.	Birth center or hospital
Between reimbursement and buildings and meeting the regs, and sustainability, how do you sustain your bottom line, especially if you accept Medicaid, which a lot of birth centers don't.	Birth center or hospital
**Barrier 3: Low supply of midwives**	
I think part of it is staffing, like I can't seem to find midwives. So if I could find enough midwives, I could double the practice.	Birth center or hospital
We just don't have enough midwives, that's a huge, huge part of the problem; we can't even begin to plan for more birth centers if we don't increase the number of midwives that are available to support them.	Birth center or hospital
Oh my God, I would hire a thousand midwives tomorrow, if they were lined up at my door.	Birth center or hospital
**Barrier 4: Low but growing patient demand**	
I don't think any one birth center is putting up numbers that would say, man, we really need to create more birth centers in New Jersey.	Birth center or hospital
It's just been very disappointing having to tell people I'm sorry, you can't birth at the birth center… You know, to not be able to offer that care to every single birthing person that desires it and is low risk.	Birth center or hospital
I'm not sure if folks really know about [birth centers]. I think that we, even as a health plan, could do better in terms of marketing that.	Health insurance or Medicaid
It's hard because I just told you how we don't have any money. And then the first thing to go would be our marketing money. So we have to figure out how we… battle these huge monolith health care systems that have millions of dollars for advertising.	Birth center or hospital
This medicalized notion of birth being just a very dangerous time for a woman has colored much of the population's interest or concern with birthing in a birth center where they think like, oh, something's gonna go terribly wrong… The only choice is to birth in a hospital.	Birth center or hospital
I think that there is like long‐term trauma related to [medical racism] that makes it very difficult for someone to birth in a hospital in a way that they would feel respected and trusted and cared for. And I think that we've seen that perpetuated. You know, not even historically, there's just systemic racism baked into our medical system. And so, I think the idea of accessing a birth center is potentially more comforting and supportive for individuals of color in that state.	Policy‐adjacent philanthropy or research
I think the demand is there. And I think, especially among Black women, I've seen a big shift, and Black women are coming to us [a birth center]. They're consuming all of this scary content on the internet and the news, it's very scary, to hear all of these statistics that you're a part of. And so, I think they find us [birth centers] more… More people are educating themselves about these problems.	Birth center or hospital
Patients are happy out of the hospital and they discover that they can have birth safely outside the hospital.	Birth center or hospital

### Direct Financial Barriers

#### Inadequate Facility Reimbursement

Low facility reimbursement rates were described by nearly all participants as the main barrier to expanded access and birth center success in New Jersey. As a state official interviewee stated, “the reimbursement rates aren't the best for Medicaid, it's sometimes more financially sound or more financially beneficial [for a facility] to just not accept Medicaid.” Providers who were interviewed understood the value in serving Medicaid patients but found the rates too low for sustainable operations. “We don't have contracts with all the Medicaid MCOs because some of them are offering us such low, low reimbursement that it wouldn't even pay for, you know, the sheets” (birth center‐hospital participant). This primary barrier was further summarized through a policy lens “I think if the regs would change… so that all birth centers could afford to accept Medicaid, I would like to see that so that everyone has access” (birth center‐hospital participant).

#### Startup and Operating Costs

The other direct financial barrier for birth centers is the startup and ongoing operating costs for birth centers (eg, licensing fees, malpractice insurance). Many midwifery practices inherently view birth as a normal physiologic process, provide regular home birth services, and attend births in a variety of nonclinical spaces; for them, meeting state facility requirements can be an economic and process burden. As described by a birth center‐hospital participant:

With the New Jersey regs, you can't like… like older birth centers in New Jersey were in an old Victorian home… that would never fly now with the ambulatory regulations. Like you have to have the hallway be a certain size, it costs so much money just to be able to meet the ambulatory requirements. We're not like other states where we could just have a birth center in a home anymore.

The startup costs for a new facility are significant. Building or retrofitting to become a state‐regulated birth center requires architecture and design services, in addition to the equipment and staffing. One birth center‐hospital interviewee described the economic toll: “This project was 5 million, who has that kind of money? Certainly not a midwifery practice.” Combining the startup costs, ongoing fees, and low insurance reimbursement rates creates financial instability for many birth centers. Other sources of funding are needed for sustainable operations, as highlighted by a birth center‐hospital interviewee: “Between reimbursement and buildings and meeting the regs, and sustainability, how do you sustain your bottom line, especially if you accept Medicaid, which a lot of birth centers don't.”

These direct financial barriers have immediate impact on birth center operations and accessibility to birthing people of color and those with low income. The next set of indirect barriers are secondary factors that, if resolved, would lead to increased patient access and use and therefore more sustainable birth center operations.

### Indirect Financial Barriers

#### Low Supply of Midwives

A major barrier raised by nearly all interviewees was the need for more midwives who can practice in birth centers. The imbalanced supply and demand for midwives was best summarized by several interviewees from the birth center‐hospital sector:

I think part of it is staffing, like I can't seem to find midwives. So if I could find enough midwives, I could double the practice.

We just don't have enough midwives, that's a huge, huge part of the problem, we can't even begin to plan for more birth centers if we don't increase the number of midwives that are available to support them.

Oh my God, I would hire a thousand midwives tomorrow, if they were lined up at my door.

Birth center participants also noted the desire to recruit and hire midwives and staff of color, which they describe as a challenge now. Increasing midwifery availability and diversity is necessary for birth centers, which are serviced exclusively by midwives, in order to expand operations and appeal to the diverse New Jersey population.

#### Low but Growing Patient Demand

An interesting feature of the interview results were the conflicting perspectives regarding awareness of and demand for birth center services. One participant from the birth center‐hospital sector stated, “I don't think any one birth center is putting up numbers that would say, man, we really need to create more birth centers in New Jersey.” Conversely, another from the same sector reflected on turning patients with Medicaid coverage away from care: “It's just been very disappointing having to tell people I'm sorry, you can't birth at the birth center… You know, to not be able to offer that care to every single birthing person that desires it and is low risk.” If they have enough staffing, the birth centers all reported having capacity for more patients, but patient awareness of services varied. An interviewee representing the health insurance sector said, “I'm not sure if folks really know about [birth centers]. I think that we, even as a health plan, could do better in terms of marketing that.” Marketing for birth centers and midwifery practices, however, has not typically been a priority. It also requires funds that birth centers may not be able to afford to expend, as a participant from the birth center‐hospital sector explained:

It's hard, because I just told you how we don't have any money. And then the first thing to go would be our marketing money. So we have to figure out how we… battle these huge monolith healthcare systems that have millions of dollars for advertising.

Similarly, participants described conflicting patient perspectives on the safety of birth centers, further limiting demand. Participants shared perspectives that they hear from patients as well as the social context around birth. On one hand, hospitals are viewed as safer:

This medicalized notion of birth being just a very dangerous time for a woman has colored much of the population's interest or concern with birthing in a birth center where they think like, oh, something's gonna go terribly wrong… The only choice is to birth in a hospital. (Birth center‐hospital participant)

Birth center outcomes indicate that they are a safe and appropriate setting to support physiologic birth. Participants noted this as being particularly important for women of color:

I think that there is like long‐term trauma related to [medical racism] that makes it very difficult for someone to birth in a hospital in a way that they would feel respected and trusted and cared for. And I think we've seen that perpetuated. You know, not even historically, there's just systemic racism baked into our medical system. And so, I think the idea of accessing a birth center is potentially more comforting and supportive for individuals of color in the state. (Policy‐adjacent philanthropy or research participant)

Similarly, a birth center‐hospital participant relayed the demand for safe out‐of‐hospital birth among Black birthing patients:

I think the demand is there. And I think, especially among Black women, I've seen a big shift, and Black women are coming to us [a birth center]. They're consuming all of this scary content on the internet and the news, it's very scary, to hear all of these statistics that you're a part of. And so, I think they find us [birth centers] more… More people are educating themselves about these problems.

Although there are few birth center births, these are increasing, in part due to the COVID‐19 pandemic, which led to shifts both in demand and perception of safety. As one participant from the birth center‐hospital sector described, “patients are happy out of the hospital and they discover that they can have birth *safely* outside the hospital.”

## DISCUSSION

The direct financial barriers for birth centers to expand access to pregnant people with low income in New Jersey, including inadequate Medicaid facility reimbursement rates, and startup and ongoing operating costs, were referenced by nearly all interview participants. New Jersey has one of the lowest Medicaid reimbursement rates for birth center facility fees at $1300 for on‐site birth in 2023 ($500 if hospital transfer occurs after admission),^31^ compared with a national average of just above $1900 in 2011, the latest data available and certainly higher now.^32^ Furthermore, the reimbursement rate is a state‐legislated dollar amount, although there are grassroots efforts to remove the facility fee from legislation. In contrast, Massachusetts recently updated their birth center facility fee reimbursement rate to more than $6000, making it 4.6 times higher than New Jersey's.^33^ As of November 2023, 3 of the 4 freestanding birth centers in New Jersey were Medicaid‐accepting facilities, although with coverage limitations. Being a fully Medicaid‐reimbursable facility (contracting with all MCOs) would further expand access to more people with low income in New Jersey and, in turn, provide more choice to Medicaid‐covered pregnant people. But with such low facility reimbursement, especially when transfers occur, birth centers are incentivized to limit or screen out even low‐risk pregnant people with Medicaid coverage. There was evidence of this disincentivizing in the data, as well as in the literature.^15^

In New Jersey, birth center facilities are regulated and licensed to operate under the broader licensing standards for ambulatory care facilities.^34^ Initial licensing application fees are $1750 for a birth center and $750 for annual renewals, with additional fees for extra services and inspection fees.^34^ Insurance costs, although not unique to midwifery, are an additional expense. Combined, these costs present barriers to birth center sustainability and patients’ access to this birth setting.

The indirect barriers, including low supply of midwives and low patient demand, were prominent in the interview data but less directly affect immediate finances of birth centers. For example, a lack of available midwives prevents birth centers from providing services to their full facility capacity. There are recent efforts to expand midwifery training in the state, with Rutgers School of Nursing launching a Master of Science Nursing program in 2024, in addition to their current Doctorate of Nursing in Midwifery. But there is still a need to develop preceptorship and career pathways that fully integrate midwifery into the health care system. Supply of midwifery providers is also linked to inadequate practice and payment structures. Certified professional midwives do not have independent practice authority in New Jersey, unlike other states such as Rhode Island where independent practice authority allows for innovative funding models.^35^ Independent practice authority is a primary initiative of the American College of Nurse‐Midwives New Jersey State Affiliate, which would increase the feasibility and availability for all midwives to practice in community birth settings. Until recently, midwives had a lower reimbursement rate than physicians for birth services; a 2023 update to New Jersey Medicaid guidelines increased the midwifery rate from 95% to 100% of the physician rate for certain maternity‐related services.^36^ Although an improvement, this pay parity has minimal impact, as it is not mandated for MCOs, only Medicaid's fee‐for‐service program.

Patient awareness and perceived safety of birth centers are conceptually linked, with both correlating to reduced demand for birth center care. Labor and birth pose potential maternal and fetal risks regardless of the setting.^6^ Despite evidence of birth center safety and culturally centered care, doubts and misconceptions remain.^2^, ^32^ The increased awareness and use during the COVID‐19 pandemic points to the need for birth centers to both market themselves differently and prepare for fluctuating demand. It remains to be seen if the growing demand for birth centers continues beyond the pandemic years. Limited awareness and conflicting or negative perceptions of birth center safety keep demand and use lower than capacity would otherwise allow.

### Implications and Recommendations

The barriers to access presented here can be redefined as solutions or facilitators to care. Reframing the barriers into facilitators results in a series of concrete policy recommendations. Medicaid reimbursement rates for the birth center facility fee should be assessed regularly and increased accordingly, rather than codified in rigid state legislation. MCO rates and commercial insurance would likely follow suit. Other financial incentives like education initiatives for student midwives and training in birth centers are also recommended.^37^, ^38^ Licensing and ambulatory care regulations could reconsider add‐on and renewal fees to lessen the burden on birth centers. Collaborative relationships between birth centers, health systems, and insurance providers could generate new patient interest by highlighting safe and integrated transfer networks, with value‐based payments for successful transfer of care. Practice‐based solutions, like incentives for midwives of color in the workforce, could improve racial and cultural concordance with patients, which is known to improve birth outcomes.^39^ Provider‐patient concordance may also be linked to patient selection of a provider and improved health care experience and satisfaction.^40^, ^41^, ^42^ Overall, improving on these financial barriers can help expand access to birth centers, result in fewer reports of discrimination by birthing individuals, and provide choice and autonomy in care.^32^, ^42^

### Strengths and Limitations

Although this research focused on the birth center ecosystem in New Jersey, many of the themes and conclusions are generalizable to other states and different health care settings. This study also validates similar research. Findings for New Jersey both confirm and build on the challenges identified in the Strong Start evaluation, which also identified insurance reimbursement rates as a leading barrier.^5^, ^8^ A limitation is the absence of personal accounts from the population of interest (ie, birthing people with lower incomes and those of color). Courtot and colleagues incorporated participation from this population in the national study of Strong Start birth centers, with which findings from this study align.^8^ An important area of further research would be to hear directly from individuals in this populations’ views of the barriers to access, and to triangulate those findings with the provider‐ and policy‐oriented perspectives included here.

## CONCLUSION

Freestanding birth centers are a proven safe and effective birth setting for individuals with low‐risk pregnancies, but financial barriers prevent widespread accessibility in New Jersey and elsewhere. Medicaid reimbursement rates are a primary barrier, along with startup and operating costs, midwifery workforce availability and payment structures, patient awareness, and general knowledge of birth center safety. Reimbursement and operating costs can be improved through state legislative action to adjust rates and costs more appropriate to birth center operations. Legislative efforts have promise to improve the financial sustainability of birth centers, increasing sustainability and availability to all birthing people in New Jersey and particularly people of color and those with low income. Additional solutions like independent midwifery practice and reimbursement parity are already underway in New Jersey through enacted or proposed legislative updates. With New Jersey's other efforts around equitable maternal health care and access, the state is well positioned to enact critical legislative and practice changes that would serve as exemplary solutions for other states to follow suit, improving birth center sustainability and access nationwide. States with similar demographic and geographic profiles could benefit from New Jersey's access challenges for similar policy and practice improvements.

## CONFLICT OF INTEREST

The authors have no conflicts of interest to disclose.

## Supporting information


**Appendix S1**. Standards for Reporting Qualitative Research (SRQR)*
